# Elucidating knowledge and beliefs about obesity and eating disorders among key stakeholders: paving the way for an integrated approach to health promotion

**DOI:** 10.1186/s12889-019-7971-y

**Published:** 2019-12-16

**Authors:** Bianca Bullivant, Aaron R. Denham, Clare Stephens, Rebecca E. Olson, Deborah Mitchison, Timothy Gill, Sarah Maguire, Janet D. Latner, Phillipa Hay, Bryan Rodgers, Richard J. Stevenson, Stephen Touyz, Jonathan M. Mond

**Affiliations:** 1 Boden Institute of Obesity, Nutrition, Exercise and Eating Disorders, Faculty of Medicine, The University of Sydney, Sydney, NSW 2006 Australia; 2 Macquarie University, Sydney, New South Wales Australia; 3 The University of Queensland, St. Lucia, Queensland Australia; 4 Western Sydney University, Penrith, New South Wales Australia; 5 University of Hawaii at Manoa, Honolulu, Hawaii USA; 6 Australian National University, Canberra, Australian Capital Territory Australia; 7 University of Tasmania, Launceston, Tasmania Australia

**Keywords:** Eating disorders, Obesity, Integration, Health promotion, Stakeholders

## Abstract

**Background:**

Understanding the knowledge and beliefs of key stakeholders is crucial in developing effective public health interventions. Knowledge and beliefs about obesity and eating disorders (EDs) have rarely been considered, despite increasing awareness of the need for integrated health promotion programs. We investigated key aspects of knowledge and beliefs about obesity and EDs among key stakeholders in Australia.

**Methods:**

Using a semi-structured question guide, eight focus groups and seven individual interviews were conducted with 62 participants including health professionals, personal trainers, teachers and consumer group representatives. An inductive thematic approach was used for data analysis.

**Results:**

The findings suggest that, relative to obesity, EDs are poorly understood among teachers, personal trainers, and certain health professionals. Areas of commonality and distinction between the two conditions were identified. Integrated health promotion efforts that focus on shared risk (e.g., low self-esteem, body dissatisfaction) and protective (e.g., healthy eating, regular exercise) factors were supported. Suggested target groups for such efforts included young children, adolescents and parents.

**Conclusions:**

The findings indicate areas where the EDs and obesity fields have common ground and can work together in developing integrated health promotion programs.

## Background

Obesity and EDs are significant public health problems characterised by substantial impairment in quality of life, high burden of disease more generally, and resistance to treatment [1, 2]. Current trends suggest that eating and weight-related health issues are increasing and are likely to present public health challenges for the next few decades [3, 4]. Given the adverse physical and mental health outcomes associated with these conditions [1, 2, 5]. continued efforts to reduce this burden are essential [6].

Overweight and obesity are defined by the World Health Organization (WHO) as an excessive fat accumulation that presents a risk to an individuals’ health [7]. They are typically measured using the Body Mass Index (BMI), a population-level measure of weight relative to height. Adults with a BMI ≥ 25.0 and < 30.0 are classified as overweight, while those with a BMI ≥30 are classified as obese [5, 7]. Additionally, obesity is divided into mild (BMI ≥ 30.0 and < 34.9), moderate (BMI ≥ 35.0 and < 39.9) and severe (BMI ≥ 40.0) categories. Obesity is often, but not always, associated with physical health impairment, the degree of this impairment being related to the degree of overweight [2, 5]. It is not classified as a psychiatric disorder [8] and is typically not associated with mental health impairment in the absence of mediating factors, such as body dissatisfaction and physical health impairment [9, 10].

EDs are a disparate group of mental health problems that are characterised by body image disturbance in the form of extreme concerns about weight or shape and the regular occurrence of eating and/or weight-control behaviours that are deemed to be “pathological” or “disordered” [11]. There are several types of EDs, including anorexia nervosa (AN), bulimia nervosa (BN), binge eating disorder (BED) and variants of these disorders such as “purging disorder” and “night eating syndrome” [11, 12]. Pathological behaviours associated with these disorders include binge eating, purging (self-induced vomiting and/or misuse of laxatives or diet pills), extreme dietary restriction, and excessive exercise [13]. EDs are by definition associated with marked impairment in mental health and may also be associated with physical health impairment, where body weight is very low or very high and/or when frequent use of purging behaviours is present for example [2, 12].

To date, public health programs for obesity and EDs have remained largely disparate, despite increasing awareness of the overlap between these conditions and the fact that treatments for both conditions remain sub-optimal [14–16]. Various shared risk (e.g., dieting and weight-control behaviours, body dissatisfaction, low self-esteem, depression symptoms and anxiety, media and marketing exposure) and protective (e.g., enjoying physical activity, high self-esteem, positive body image) factors for the respective conditions have been identified [17–19]. Furthermore, there is extensive overlap between obesity and EDs in that these conditions can co-occur in the same individual and individuals can move from one condition to another over time [3, 20]. In particular, individuals experiencing overweight or obesity are at a higher risk of disordered eating and EDs than the general population [21], and individuals with BED have particularly high rates of overweight and obesity [22].

Despite these commonalities, dialogue between the obesity and ED fields is rare and collaboration in the development of integrated prevention and health promotion programs rarer still. Reasons for this likely include the different backgrounds of prevention and health promotion researchers in their respective fields – obesity primarily in public health, EDs primarily in clinical psychology – (cf. [23]) and the fact that certain obesity health promotion messages (e.g., increased focus on body weight and/or diet) may be seen to conflict with certain EDs health promotion messages (e.g., reduced focus on body weight and/or diet) [6, 24]. Indeed, one of the consequences of the lack of collaboration between researchers in these respective fields is that little consideration has been given to the possibility that the messages being delivered to the public from these fields may be conflicting or at least confusing.

An additional impediment to collaboration between the fields, in our view, is that there is a paucity of information concerning knowledge of and beliefs about obesity and EDs – and the overlap between these conditions – among key stakeholders. In the field of health communication, it is generally accepted that improved understanding of the knowledge and beliefs of target audiences is conducive to the development of more effective message communication [25, 26]. In recent years, there has been considerable interest in public perceptions of the framing of obesity prevention messages (e.g., [27]) and the importance of elucidating public knowledge and beliefs about EDs as a platform for health education efforts is increasingly recognised [23, 28]. Both obesity and ED are highly stigmatised conditions [29–32] and stigmatising attitudes and beliefs would be expected to manifest themselves in distinct ways among different stakeholders, including healthcare providers [33, 34]. Importantly, there is growing support for “bottom-up” approaches to public health interventions for both obesity and EDs, in which public opinion is solicited in the course of policy development [28, 35].

Information from other key stakeholders, such as health professionals and educators, is also important, however, both in promoting cross-sectoral collaboration and in informing the conduct of proposed interventions [36, 37]. For example, information from key stakeholders may be helpful in developing a set of potential integrated health promotion messages for obesity and EDs, the perceived acceptability and persuasiveness of which in different demographic subgroups might then be systematically examined (e.g., [27, 38]). While the opinions of key stakeholders, most notably school educators and parents, concerning the design and conduct of interventions for obesity (e.g., [39, 40]) and EDs (e.g., [41]) have been examined, these studies have typically been confined to the opinions of one or a small number of stakeholders. Further, this research has been confined to studies of stakeholder opinions concerning interventions for obesity and ED *considered separately*.

With these considerations in mind, the goal of the current study was to elucidate knowledge and beliefs concerning obesity and EDs – and the relation between these conditions – among a broad range of stakeholders (e.g., health professionals, teachers, academics, and obesity and ED consumer group representatives). The aspects of participants’ knowledge and beliefs considered included the seriousness of and relative importance of obesity and EDs as public health problems, the links between obesity and EDs, the desirability and feasibility of an integrated approach to prevention, specific health promotion messages likely to be most effective in the context of an integrated approach, the optimal methods for delivering these messages and barriers to integrated health promotion strategies.

## Methods

### Participants

The study sample was comprised of 62 Australian individuals who agreed to participate in focus groups and interviews. Participants were recruited based on their knowledge and expertise with weight and shape related issues. While the type and composition of stakeholder groups were determined in advance, we recruited eligible participants primarily through convenience sampling techniques. All participants were contacted via emails and relevant agencies, with an invitation to take part in the study.

Eight focus groups were initially conducted with 55 participants. The first three focus groups (focus group 1 (*n =* 6); focus group 2 (*n* = 12); focus group 3 (*n* = 5) were held during the 2015 National Eating Disorders and Obesity Conference held in the Gold Coast, Australia. These focus groups were inter-disciplinary and included a range of people, including psychologists, teachers, general practitioners, registered dietitians, nurses, public health professionals, social workers, and endocrinologists. The next five focus groups were intra-disciplinary; one focus group was conducted with teachers (*n* = 5), another with psychology academics (*n =* 4), personal trainers (*n* = 6), professionals from ED consumer/advocacy groups (Butterfly Foundation/National Eating Disorders Collaboration (NEDC); *n* = 7) and one with members of an obesity consumer/advocacy group (Obesity Support Council; *n* = 10).

Seven in-depth individual interviews were then conducted with health professionals working with individuals with obesity and/or EDs, including dietitians, psychologists, an endocrinologist and an exercise physiologist. The individuals who participated in the interviews were not involved in the focus groups. Table 1 summarises the participants’ demographic characteristics.

**Table 1:** Demographic characteristics of participants

	Focus groups	Interviews
Total number	55	7
Gender		
Male	14	2
Female	41	5
Age (years)		
18–24	6	0
25–34	18	4
35–44	11	2
45–55	14	1
Over 55	6	0
First language		
English	49	6
Other	6	1
Position		
Dietitian	5	3
Psychologist	4	2
Endocrinologist	1	1
Exercise physiologist	–	1
Personal trainer	6	–
General practitioner	3	–
Project coordinator	3	–
Social worker	1	–
Nurse	2	–
Teacher	6	–
Public health professional	3	–
Psychology academic	4	–
Butterfly Foundation/NEDC	7	–
Obesity Support Council	10	–

### Procedure

The qualitative interviews and focus groups were guided by phenomenological and interpretive theories. Phenomenological approaches prioritise participants’ experiences, folk and professional categories and models, reasoning, and meaning of illness and wellbeing (see [42]). Interpretive paradigms direct attention to in-depth descriptions and the meaning of shared experiences, categories, and metaphors (see [43]). From this, our analytic framework followed an inductive approach drawing on grounded theory [44, 45], which cultivated an analytical space permitting participant themes to emerge from the data [46].

Prior to the start of each focus group/interview, the consent form was reviewed and signed by both the participant and facilitator. The focus groups were guided by a series of semi-structured, open-ended questions that were developed based on the extant literature [14, 18, 24], and goals of the research. Questions were drafted to elicit the categories, concepts, and beliefs that participants felt were important. The guide focused on several relevant topics, such as knowledge and beliefs about the seriousness and relative importance of obesity and EDs as public health problems, beliefs about the links between obesity and EDs, beliefs about the desirability and feasibility of an integrated approach to prevention, beliefs about the specific health promotion messages likely to be most effective in the context of an integrated approach, and the optimal methods for delivering such messages (see Table 2). Both inter- and intra-disciplinary focus groups were conducted to capture a wide range of perspectives, and to see if the resultant discussion and interactions between participants revealed areas of consensus, tension, and/or disagreement.

**Table 2:** Selected focus group and interview research questions

What is your understanding of obesity?	
What is your understanding of eating disorders?	
To what extent do you believe obesity/eating disorder behaviours are public health problems requiring intervention?	
What are your beliefs regarding the links between obesity and eating disorders?	
To what extent do you believe there is a need for integrated obesity and eating disorders health promotion?	
What kind of efforts/messages do you believe would be most effective for targeting both obesity and eating disorders?	
Which modes of delivery, do you believe, are optimal for delivering integrated messages for obesity and eating disorders?	
What are some of the barriers that you can see to integrated health promotion efforts?	

Individual interviews were then scheduled with individuals representing selected professions in order to gain additional insight into their perspectives. The interviews allowed these participants to express their views on these topics outside of a group setting and its limitations [47]. The same questions asked of focus group participants were asked during these interviews, along with additional questions exploring participants’ understanding of disordered eating behaviours and the barriers to integrated health promotion efforts.

The duration of focus groups was approximately 1 h, while the duration of the interviews ranged from 20 to 40 min. Every focus group and interview was audio recorded and transcribed verbatim, with identifying information (names of participants, places of employment) removed. Data collection continued until saturation of themes had been achieved [48]. Saturation was deemed to have been reached when no new themes were arising from the discussions. Immediately before the focus groups/interviews started, participants filled out a questionnaire collecting socio-demographic information. All participants received a gift card for participating in the study. Ethics approval for this study was obtained by the Macquarie University Human Ethics Committee.

### Data analysis

Inductive thematic analysis, an approach to data analysis common to the constructivist paradigm, was used to identify major themes in the textual data [49]. Two members of the research team read and re-read the transcripts to familiarise themselves with the data, identify patterns and develop the thematic codes related to the research questions [50]. The focus groups and interviews were coded using NVivo software and the codes were compared to increase intercoder reliability. The codes were integrated, assessed and used to develop the major themes. The major themes were also compared within and between the focus groups and interviews.

## Results

The participants’ knowledge and beliefs concerning EDs and obesity and their views on integrated health promotion strategies were the primary topics explored. The participants’ conversations about obesity and ED health promotion and prevention revealed both shared positions and points of disagreement. Below we address findings relating to the following themes: (a) awareness and understanding of obesity and EDs; (b) beliefs about the links between obesity and EDs; (c) attitudes towards integrated obesity and ED health promotion; and (d) beliefs about target groups (i.e., who should be targeted) in such programs .

### Awareness and understanding of obesity and EDs

Discussions began by asking participants how they defined the terms ‘obesity’ and ‘EDs’. Obesity was typically defined by participants in terms of elevated BMI (e.g. BMI > 30) or outward physical size. Participants from all focus groups and interviews identified this clinical definition of obesity and described the associated risk factors, comorbidities and health outcomes.

Participants defined EDs as mental health conditions that are centred around problematic eating behaviours. AN and BN were most commonly referenced when defining EDs. Fewer references were made to BED or other specified/unspecified feeding and EDs. ED practitioners expressed frustration about the lack of recognition surrounding BED. One psychologist remarked, “It’s actually quite neglected, the focus on anorexia and bulimia, whereas the binge eating disorder, medical doctors don’t even recognise it, so it’s very frustrating.”

Disordered eating practices were also discussed among participants and were identified as unhealthy eating practices, such as binge eating, dieting, restricting, purging and having food rules. Participants distinguished between disordered eating practices and EDs, “I think there are obvious similarities between eating disorder behaviour and eating disorders,” remarked an exercise physiologist, “particularly around poor eating habits, but eating disorders are a more severe expression of that.”

Those participants working primarily in an obesity setting, teachers, public health workers, and personal trainers, noted that they didn’t have a great depth of knowledge of EDs and that their education and training programs often did not include information on EDs. A student remarked that information on EDs has not been included in their public health course curriculum. “So, it’s interesting,” they said, “that we talk about obesity, but we don’t even necessarily talk about the binge eating that goes with obesity.”

Participants across stakeholder groups expressed concern about how the public perceives these conditions. Those working primarily within the domain of EDs described the public as unaware of the breadth of symptoms and the diversity of diagnostic labels within the larger category of EDs. For example, misperceptions that only young women are at risk and that EDs are a method for drawing attention to oneself are widespread. In terms of obesity, participants remarked on the stigma surrounding obesity from the public and health professionals, particularly around personal responsibility, labelling people with obesity as fat, less hardworking and unhealthy. Health professionals were concerned this stigma poses a barrier to people with obesity accessing health services.

Regarding the seriousness of obesity and disordered eating behaviours, many participants viewed obesity as an important public health problem. They frequently cited the high prevalence rates among adults and children, severe health comorbidities, and cost to the healthcare sector and society. It is not just the costs to the public health system, as a nurse working in an obesity setting explained, “[It is] also a cost to the family and the individual as well” in terms of financial and emotional burdens.

When compared to obesity, participants across the professional groups viewed EDs as less of a public health concern, noting how obesity leads to many other health problems, affects a greater number of people, and costs the public health sector more than EDs. There are significant public health challenges with obesity alone that linking EDs to obesity, according to a psychology academic, is a “relatively small kind of component. So, I see the obesity as the biggest issue perhaps.” However, those working in the ED field noted that an increase in not only EDs but also disordered eating behaviours is a public health concern. Increases in these behaviours were seen as a significant issue by a psychologist since they can be a “precursor or an aftereffect of an eating disorder.”

### Beliefs about the links between obesity and EDs

Across all focus groups and interviews, participants identified areas in which the obesity and EDs fields hold common ground. This generally related to shared risk factors, such as binge eating, dieting, disordered eating, unhealthy relationships with food, and low self-esteem. Shared protective factors, including healthy eating, healthy lifestyle, a good relationship with food, strong self-esteem, and social support, were also acknowledged. How the risk and protective factors and shared health outcomes are identified and often treated individually, rather than as a set of interconnected problems, was raised as an issue. As a participant from the EDs consumer/advocacy focus group commented:

I think the danger is we always see these in silos as conditions rather than looking at risk factors and protective factors. Actually, a lot of the risk and protective factors can protect against lots of different things.

Participants working with people with obesity also recognised that EDs and obesity can co-occur and are interrelated. That is, they identified how dieting and binge eating can lead to weight gain and how many individuals who are overweight or obese meet the criteria for BED. An exercise physiologist described how binge eaters might respond to messages about obesity prevention, stating:

The binge eating, even people responding to messages about obesity that they should stop eating, they should eat less, so they might be doing that type of eating and then rebound and do the opposite as well.

Participants in separate focus groups described how eating behaviours, weight, and body image issues exist on a continuum and often intersect. For instance, an individual in the ED consumer/advocacy focus group said:

I’d say there is a spectrum, there’s healthy functioning, healthy eating, and we go along the spectrum, then we come across to a space that perhaps includes disordered eating, and that can both include people who may be suffering from obesity or suffering from an eating disorder, and eventually we get into a medical diagnosis of both.

Similarly, in a separate interdisciplinary focus group, a dietitian noted:

It’s like a continuum that we’re both working together on the same end of. [It’s] important to consider the psychology of it along the way as well...you can’t address obesity issues or restriction issues or any other eating-related problems without acknowledging that there are thought processes and behaviours and feelings behind what people are doing.

Others, however, viewed these conditions as separate and were sceptical about the links between obesity and EDs. These participants noted that obesity is not always associated with disordered eating or an ED. Health professionals described the treatment of these conditions are separate. A manager at an ED support organisation stated, “I tend to split them in my mind and treat them quite separately, even though I’m very well aware that you may have an increased risk, for instance, if you have obesity.”

Mental health practitioners identified emotional or psychological issues as a key distinction between EDs and obesity. Thus, the classification and treatment of obesity and EDs were viewed as separate issues. A psychologist remarked, “I think, EDs obviously are a mental health issue, I don’t think that obesity is on its own.” Differences in opinion were apparent both across and within stakeholder groups. For example, two teachers disagreed about whether obesity has a mental health component:

It would depend on the situation of the person because some people their link to obesity might be some mental health issues. Others their link to obesity could be, for example, some cultural links. (Teacher 1).

Whereas I would say they are completely linked because I think obesity is an eating disorder in itself. (Teacher 2).

### Attitudes towards integrated obesity and ED health promotion

Across stakeholder groups, many participants expressed a need for an integrated approach to public health interventions. Greater collaboration between the obesity prevention and ED prevention fields was seen as critical in establishing core beliefs that both sides share, and which can form the basis of messaging. A project coordinator for an ED organisation noted:

It’s really important to have cross-sector collaboration...it’s important, with the potential for unintended harm in messaging from both obesity and eating disorders, that we work together to come up with health promotion campaigns which do their best to cause no harm, whilst achieving the goals that we want to achieve.

There was considerable support across stakeholder groups for integrated health promotion messages that addressed the shared risk and protective factors for both conditions. Suggested ideas for integrated messages included focusing on health and well-being, positive body image, healthy eating, strong self-esteem, eating in moderation, and regular physical activity. Some participants considered it to be particularly important that any integrated messages focus on improvements in health and not weight loss. A psychologist suggested that, “health promotion messages need to be weight neutral and positive in order to improve those messages.”

Not all participants were supportive of this approach, however. Disagreement about an integrated approach can be seen in the exchange below between a nurse working in an obesity setting and a clinical psychologist also working in an obesity prevention setting:

I’m not really sure if communities are ready to hear that message, I think that it would confuse them…I’d be very concerned that they would be conflicted and just if you look at the prevalence rates, because obesity is a far greater, more prevalent problem, I think that perhaps requires addressing first. (Nurse).

I’d respectfully disagree...I think we should be sending a single, united, strong message about, ‘this is healthy eating, this is a healthy lifestyle’ and having a healthy relationship with food. (Clinical psychologist).

Participants who were less supportive of an integrated approach preferred the use of separate messages for the different conditions. As a dietitian noted, “I think it would be best for these two conditions to be targeted separately.” These participants saw obesity and EDs as distinct issues and expressed concern that connecting the two issues for health promotion purposes would cause confusion. Participants also mentioned the stark differences between AN and obesity as an obstacle to finding common ground for integrated messages. “I think there is some overlap,” a psychology academic said, “but I think you’re going to repel people by putting it in the bunch because people don’t want to be associated.”

The feasibility of integrated health promotion efforts was questioned, even among those who were supportive of this approach. The cost of an integrated approach to health promotion program was frequently cited as a barrier. As a dietitian noted, “I guess the funding issue is probably the biggest one, always with health promotion.” Another perceived challenge for an integrated approach was the lack of collaboration between the obesity and ED fields. Participants were concerned about whether and how these fields could come together and decide on appropriate integrated messages.

Other participants supported an integrated approach to addressing obesity and EDs but one focusing on macro-level factors rather than health promotion messages. Thus, there was strong support for greater regulation of advertising directed towards children on television, advertising at sporting events, diet advertising and the availability of healthy food. A public health professional elaborated on the need for regulation:

I think health promotion messages are great, but at the end of the day, you need to make certain things affordable, certain things unaffordable, certain things available, certain things unavailable, and that will start to change things.

The need for interventions simultaneously targeting individual-level (e.g., mental health and media literacy) and macro-level (e.g., free access to fruit and vegetables in schools) determinants of health behaviours was also noted by some participants.

### Beliefs about target groups in integrated health promotion initiatives

Participants identified several appropriate target groups for integrated public health interventions, including, children, adolescents, parents and adults in the workplace. Participants also supported more education for health professionals working with vulnerable populations, including teachers, school nurses, general practitioners and personal trainers. As one personal trainer suggested, “Rather than trying to target the kids, targeting the teachers, same as parents because they’re the ones teaching them”.

Several participants supported interventions for young children. A participant from the obesity consumer/advocacy focus group suggested that “If you’re hitting them young, it has a long-term effect and that will have a generational change.” Further, several participants highlighted the need for better education programs around healthy food and eating in schools. A psychologist remarked:

In schools, I really think that’s important because you don’t necessarily have to shift their views like your starting fresh. So, I think schools would be extremely important and I mean through schools you could even educate parents potentially.

Many participants noted how children first learn about food from their parents, and how they form an emotional connection with food. Hence, there was also strong support for educating teachers and parents around healthy eating and cooking. A nurse commented: “I think education is very important and obviously it starts in the home, and the parents need to know what they should be feeding their children because a lot of parents don’t really know.”

Suggested modes for reaching these target groups included social media and smartphone applications, television, radio, the internet, advertising in community centres (e.g., shopping centres, community health centres), and via workplace and school education programs. Participants noted that a multi-component program is more likely to be effective. They also emphasised the importance of having standards around the method of delivery, as well as examining the long-term effects of public health messages.

## Discussion

Focus groups, supplemented by in-person interviews, were used to explore key stakeholders’ knowledge and beliefs about obesity, EDs and their possible intersections. The results of this study contribute to a growing area of research exploring the potential for collaboration in the development of integrated health promotion programs for obesity and EDs. The findings illustrated that while participants were universally aware of the significance of obesity as a public health problem, awareness and understanding of EDs was relatively poor among several participant groups including obesity practitioners, public health professionals, teachers and personal trainers. This included poor understanding of the different types of EDs and the behaviours comprising these disorders. Also, relative to obesity, participants viewed EDs as less of a public health concern.

In these respects, the current findings are consistent with those of previous research indicating that while the public, health professionals and other key stakeholders are generally aware of the nature and adverse consequences of obesity [51, 52], awareness and understanding of EDs among key stakeholders is relatively poor [53, 54]. This is perhaps not surprising given that public health campaigns designed to improve awareness and understanding of obesity are well established and have been extensively employed in recent years [51, 55]. By contrast, the need for public education programs for EDs is only now being recognised [23, 28]. Efforts to improve public awareness and understanding of the occurrence and adverse effects of EDs have, however, gained momentum in recent years [28, 38]. Improving awareness and understanding of EDs and eating-disordered behaviour among key stakeholders in obesity research may be particularly important moving forward. At the same time, it is apparent that misconceptions concerning obesity also exist. Thus, some participants in the current study appeared to believe that obesity is in fact a form of EDs or other mental health disorder, that obesity is invariably associated with physical and/or mental health impairment and/or that all people who meet accepted criteria for obesity need treatment of some kind.

There is growing awareness among public health researchers of the commonalities in risk, protection and intervention between obesity and EDs [14, 17]. Many of the participants in the current study identified links between these conditions, particularly in terms of shared risk and protective factors. They also identified that BED often co-existed with and shared pathways of relevance to obesity. Recognition of these commonalities is encouraging in that it suggests areas where the two fields of studies can start a dialogue. However, other participants, while acknowledging these links, were less sanguine about the potential for collaboration, describing the overlap as overstated, particularly given obesity’s lack of classification as a mental health or behavioural disorder [8].

These differences notwithstanding, there was considerable agreement among stakeholders about the need for, or at least the possibility of, an integrated approach towards health promotion. This included strong support across the stakeholder groups for integrated health promotion programs that focus on health and well-being, healthy eating, self-esteem, positive body-image, discouraging dieting, and physical activity, rather than weight loss. Targeting children, adolescents and parents in such efforts was also strongly supported. The targeting of shared risk and protective factors has been suggested as an area for greater collaboration and an effective way to offset the potential for harm [18, 24].

Support for an integrated approach was often expressed by participants out of a concern that certain obesity prevention messages may have unanticipated harmful effects. This is reflected among ED researchers, who are concerned that weight-focused obesity prevention messages may promote body dissatisfaction and eating-disordered behaviour among vulnerable individuals [14, 56]. There has also been increased concern in recent years, among advocates for the *Health at Every Size* and *Body Acceptance* movements in particular, about the adverse impact of weight-related-stigma (e.g. [6, 32]). Indeed, use of the term “obesity’ is itself seen as stigmatising and therefore inappropriate by some commentators, e.g. [57]. Obesity researchers, by contrast, are more likely to be concerned that the messages likely to be used in ED prevention programs, such as those encouraging body acceptance, may detract from obese individuals’ motivation to change diet and exercise behaviour [58, 59]. These considerations highlight, first, the need to include assessment of potential adverse outcomes of interventions for both obesity and EDs [60, 61]; and second, the challenges inherent in developing integrated health promotion messages that are likely to be acceptable to all stakeholders [14, 24].

Concerns about the feasibility of an integrated approach towards health promotion for obesity and EDs were expressed by some participants, including concerns about the lack of collaboration that currently exists between the respective fields. As has been noted, the different backgrounds and training of health promotion researchers in the respective fields has proven problematic in terms of collaborative efforts. While interventions for obesity have originated primarily from the public health domain, interventions for EDs have tended to originate from the field of clinical psychology. This has led to a lack of dialogue and, at times, ill-feeling [23, 60]. Acknowledgement and open discussion of these differences and their implications for the development of integrated programs will be critical if the two fields are going to work together moving forward. We hope that the findings presented here provide an incentive for discussion of this kind.

### Limitations and future directions

At least three limitations of the current research should be noted. First, the extent to which the findings can be generalised to all relevant groups is unclear. Although a broad range of stakeholders in the obesity and/or ED fields was represented, we may not have captured the full range of experiences. Further, it is possible that individuals with certain attitudes and beliefs, including stigmatising attitudes and beliefs, were over-represented among individuals who agreed to participate in the study. Alternatively, or in addition, the findings may have been biased by a perceived need to express socially desirable opinions among some participants. However, the repeated emergence of key themes within and across focus groups and interviews suggests that the most salient ideas were captured. Second, the recruitment approach did not permit stratification of findings according to demographic or other characteristics likely to be of interest (e.g., gender, age). Finally, additional information which could describe the participants in more detail (e.g. their level of experience working with or their attitudes towards people with these conditions) was not collected. Strengths of the research included, in addition to the recruitment of a broad range of stakeholders, the collection of data until saturation of themes was achieved and the use of two investigators to code themes. Perhaps most importantly, the current research was novel in soliciting stakeholder beliefs concerning obesity and EDs as these conditions are seen to relate to each other rather than beliefs concerning either condition alone.

The current findings may have implications for both health practitioners and health promotion researchers. For health practitioners working in the obesity setting, better training and education around EDs appears to be needed, particularly around the shared risk and protective factors for overweight/obesity and disordered eating/EDs. For practitioners in both fields, it may be important to consider looking at EDs and obesity as interconnected conditions and to review preconceptions about these conditions that may be embedded in current professional training programs. At the same time, it is important to be aware of the potential for stigmatisation and undue pathologising or “medicalisation” – of both ED and obesity [62], and the need to promote holistic frameworks when assessing and addressing the causative factors, perceptions of risk and responsibility, and potential solutions [63].

Having multidisciplinary input into curriculum development and teaching of health professional programs may be helpful in bringing about change of this kind and in pre-empting some of the challenges inherent in promoting greater collaboration between ED and obesity researchers referred to above. For health promotion researchers, the findings highlight the need for, and provide a platform for, a more collaborative approach to intervention development. As has been suggested, researchers across the respective fields will need to work together to develop a set of integrated health promotion messages that are acceptable to all key stakeholders. The perceived acceptability and persuasiveness of these messages among the public should then be examined. A potential benefit of such collaboration is greater coherence of messages being communicated about various eating and weight-related problems [23]. Integrated health promotion programs also have the potential to reach a wider audience and could be more cost-effective than implementing separate programs [14].

## Conclusion

Findings from this study contribute to growing interest in the integration of public health interventions for obesity and EDs. They uncovered previously unknown aspects of stakeholders’ knowledge and beliefs about these conditions, particularly around the awareness and understanding of EDs. The findings indicate areas of convergence and divergence among researchers and health professionals from the different fields and provide a platform for greater collaboration moving forward.

## Data Availability

Data can be made available from the corresponding author on reasonable request.

## Abbreviations

AN: Anorexia nervosa
BED: Binge eating disorder
BMI: Body mass index
BN: Bulimia nervosa
ED: Eating disorder
NEDC: National Eating Disorder Collaboration
WHO: World Health Organization

## References

[1] Deloitte Access Economics. Paying the Price: The economic and social impact of eating disorders. Melbourne: Butterfly Foundation; 2012. Available at: http://thebutterflyfoundation.org.au/wp-content/uploads/2012/12/Butterfly_Report.pdf.

[2] Mond JM, Hay PJ, Rodgers B, Owen C (2009). Comparing the health burden of eating-disordered behavior and overweight in women. J Women's Health.

[3] da Luz FQ, Sainsbury A, Mannan H, Touyz S, Mitchison D, Hay P (2017). Prevalence of obesity and comorbid eating disorder behaviors in South Australia from 1995 to 2015. Int J Obes.

[4] Walls HL, Magliano DJ, Stevenson CE, Backholer K, Mannan HR, Shaw JE (2012). Projected progression of the prevalence of obesity in Australia. Obesity.

[5] Mond JM, Baune BT (2009). Overweight, medical comorbidity and health-related quality of life in a community sample of women and men. Obesity.

[6] Macpherson-Sánchez AE (2015). Integrating fundamental concepts of obesity and eating disorders: implications for the obesity epidemic. Am J Public Health.

[7] World Health Organization. Obesity and overweight [Internet]*.* Geneva: World Health Organization; 2018 [cited 2018 March 6]. Available from: http://www.who.int/mediacentre/factsheets/fs311/en/

[8] Marcus MD, Wildes JE (2012). Obesity in DSM-5. Psychiatr Ann.

[9] Gall K, Van Zutven K, Lindstrom J, Bentley C, Gratwick-Sarll K, Harrison C, Lewis V, Mond J (2016). Obesity and emotional well-being in adolescents: roles of body dissatisfaction, loss of control eating, and self-rated health. Obesity.

[10] Van Zutven K, Mond J, Latner J, Rodgers B (2015). Obesity and psychosocial impairment: mediating roles of health status, weight/shape concerns and binge eating in a community sample of women and men. Int J Obes.

[11] American Psychiatric Association (2013). Diagnostic and statistical manual of mental disorders: DSM-5.

[12] Hay P, Mitchison D, Collado AE, González-Chica DA, Stocks N, Touyz S (2017). Burden and health-related quality of life of eating disorders, including avoidant/restrictive food intake disorder (ARFID), in the Australian population. J Eat Disord.

[13] Bentley C, Gratwick-Sarll K, Harrison C, Mond J (2015). Sex differences in psychosocial impairment associated with eating disorder features in adolescents: a school-based study. Int J Eat Disord..

[14] Sanchez-Carracedo D, Neumark-Sztainer D, López-Guimerà G (2012). Integrated prevention of obesity and eating disorders: barriers, developments and opportunities. Public Health Nutr.

[15] Levine MP, Smolak L. Prevention of negative body image, disordered eating, and eating disorders: an update. In S. Wonderlich, J. E. Mitchell, M. de Zwaan, & H. Steiger (Eds.), *Annual Review of Eating Disorders: Pt.1.* CRC Press; 2018. p. 1–14.

[16] Peirson L, Fitzpatrick-Lewis D, Morrison K, Ciliska D, Kenny M, Usman Ali M, Raina P (2015). Prevention of overweight and obesity in children and youth: a systematic review and meta-analysis. CMAJ Open.

[17] Goldschmidt AB, Wall M, Choo THJ, Becker C, Neumark-Sztainer D (2016). Shared risk factors for mood-, eating-, and weight-related health outcomes. Health Psychol.

[18] Haines J, Kleinman KP, Rifas-Shiman SL, Field AE, Austin SB (2010). Examination of shared risk and protective factors for overweight and disordered eating among adolescents. Arch Pediatr Adolesc Med.

[19] Levine MP, Smolak L (2015). The role of protective factors in the prevention of negative body image and disordered eating. Eat Disord.

[20] Field AE, Sonneville KR, Micali N, Crosby RD, Swanson SA, Laird NM (2012). Prospective association of common eating disorders and adverse outcomes. Pediatrics.

[21] Darby A, Hay P, Mond J, Rodgers B, Owen C (2007). Disordered eating behaviours and cognitions in young women with obesity: relationship with psychological status. Int J Obes.

[22] Hudson JI, Hiripi E, Pope HG, Kessler RC (2007). The prevalence and correlates of eating disorders in the National Comorbidity Survey Replication. Biol Psychiatry.

[23] Mond JM (2014). Eating disorders “mental health literacy”: an introduction. J Ment Health.

[24] Neumark-Sztainer D (2012). Integrating messages from the eating disorders field into obesity prevention. Adolesc Med State Art Rev.

[25] Noar SM, Grant Harrington N, Van Stee SK, Shemanski AR (2011). Tailored health communication to change lifestyle behaviors. Am J Lifestyle Med.

[26] Rimer BK, Kreuter MW (2006). Advancing tailored health communication: a persuasion and message effects perspective. J Commun.

[27] Puhl R, Luedicke J, Peterson JL (2013). Public reactions to obesity-related health campaigns: a randomized controlled trial. Am J Prev Med.

[28] Mond JM (2016). Optimizing prevention programs and maximizing public health impact are not the same thing. Eat Disord.

[29] Star A, Hay P, Quirk F, Mond J (2015). Perceived discrimination and favourable regard toward underweight, normal weight and obese eating disorder sufferers: implications for obesity and eating disorder population health campaigns. BMC Obesity.

[30] Thille P, Friedman M, Setchell J (2017). Weight-related stigma and health policy. CMAJ.

[31] Griffiths S, Mond JM, Murray SB, Touyz S (2015). The prevalence and adverse associations of stigmatization in people with eating disorders. Int J Eat Disord.

[32] Tomiyama AJ, Carr D, Granberg EM, Major B, Robinson E, Sutin AR, Brewis A (2018). How and why weight stigma drives the obesity ‘epidemic’and harms health. BMC Med.

[33] Alberga AS, Pickering BJ, Alix Hayden K, Ball GD, Edwards A, Jelinski S, Nutter S, Oddie S, Sharma AM, Russell-Mayhew S (2016). Weight bias reduction in health professionals: a systematic review. Clin Obes.

[34] Morgan AJ, Reavley NJ, Jorm AF, Beatson R (2016). Experiences of discrimination and positive treatment from health professionals: a national survey of adults with mental health problems. Aust N Z J Psychiatry.

[35] Huang TT, Cawley JH, Ashe M, Costa SA, Frerichs LM, Zwicker L (2015). Mobilisation of public support for policy actions to prevent obesity. Lancet.

[36] Dennis S, Hetherington SA, Borodzicz JA, Hermiz O, Zwar NA (2015). Challenges to establishing successful partnerships in community health promotion programs: local experiences from the national implementation of healthy eating activity and lifestyle (HEAL™) program. Health Promot J Austr.

[37] Pettigrew S, Pescud M, Donovan RJ (2012). Stakeholder support for school food policy expansions. Health Educ Res.

[38] McLean SA, Paxton SJ, Massey R, Hay PJ, Mond JM, Rodgers B (2016). Identifying persuasive public health messages to change community knowledge and attitudes about bulimia nervosa. J Health Commun.

[39] Clarke JL, Pallan MJ, Lancashire ER, Adab P (2015). Obesity prevention in English primary schools: headteacher perspectives. Health Promot Int.

[40] Della Torre SB, Akre C, Suris JC (2010). Obesity prevention opinions of school stakeholders: a qualitative study. J Sch Health.

[41] Varnado-Sullivan PJ, Parr F, O’Grady MA, Savoy S (2013). Educators’ views of eating disorder prevention programs. Eat Weight Disord.

[42] Kumar A (2012). Using phenomenological research methods in qualitative health research. J Hum Sci.

[43] Fade S (2004). Using interpretative phenomenological analysis for public health nutrition and dietetic research: a practical guide. Proc Nutr Soc.

[44] Strauss AL (1987). Qualitative analysis for social scientists.

[45] Strauss AL, Corbin J (1990). Basics of qualitative research.

[46] Ryan GW, Bernard HR (2003). Techniques to identify themes. Field Methods.

[47] Bernard HR. Research methods in anthropology: Qualitative and quantitative approaches. 6th ed. Rowman & Littlefield; 2017.

[48] Guetterman T. Descriptions of sampling practices within five approaches to qualitative research in education and the health sciences. Forum Qual Soc Res. 2015;16(2). 10.17169/fqs-16.2.2290.

[49] Braun V, Clarke V (2006). Using thematic analysis in psychology. Qual Res Psychol.

[50] Joffe H, Yardley L, Marks DF, Yardley L (2004). Content and thematic analysis. Research methods for clinical and health psychology.

[51] Olds T, Thomas S, Lewis S, Petkov J (2013). Clustering of attitudes towards obesity: a mixed methods study of Australian parents and children. Int J Behav Nutr Phys Act.

[52] Story MT, Neumark-Stzainer DR, Sherwood NE, Holt K, Sofka D, Trowbridge FL (2002). Management of child and adolescent obesity: attitudes, barriers, skills, and training needs among health care professionals. Pediatrics.

[53] Darby AM, Hay PJ, Mond JM, Quirk F (2012). Community recognition and beliefs about anorexia nervosa and its treatment. Int J Eat Disord.

[54] Hay P, Darby A, Mond J (2007). Knowledge and beliefs about bulimia nervosa and its treatment: a comparative study of three disciplines. J Clin Psychol Med Settings.

[55] Gollust SE, Niederdeppe J, Barry CL (2013). Framing the consequences of childhood obesity to increase public support for obesity prevention policy. Am J Public Health.

[56] Schwartz MB, Henderson KE (2009). Does obesity prevention cause eating disorders?. J Am Acad Child Adoles Psychiatry.

[57] Meadows A, Daníelsdóttir S (2016). What's in a word? On weight stigma and terminology. Front Psychol.

[58] O'Dea JA (2005). School-based health education strategies for the improvement of body image and prevention of eating problems: an overview of safe and successful interventions. Health Educ.

[59] Russell-Mayhew S (2013). Interventions for the concurrent prevention of eating disorders and obesity: challenges, opportunities and implications. Can J Diabetes.

[60] Hill AJ (2007). Obesity and eating disorders. Obes Rev.

[61] Mond J, Mitchison D, Latner J, Hay P, Owen C, Rodgers B (2013). Quality of life impairment associated with body dissatisfaction in a general population sample of women. BMC Public Health.

[62] Conrad P (2007). The Medicalization Soc.

[63] Marmot M, Wilkinson RG (2006). Social determinants of health.

